# Climate Change and Livestock Welfare in the Alps: A Comprehensive Review

**DOI:** 10.3390/ani15243578

**Published:** 2025-12-12

**Authors:** Paolo Cornale, Roberto Senatore, Luca Maria Battaglini, Mario Baratta

**Affiliations:** 1 Department of Agricultural, Forest and Food Sciences, University of Turin, Largo Paolo Braccini 2, Grugliasco, 10095 Turin, Italy; paolo.cornale@unito.it (P.C.); r.senatore@unito.it (R.S.); luca.battaglini@unito.it (L.M.B.); 2 Department of Chemistry, Life Sciences and Environmental Sustainability, University of Parma, Parco Area delle Scienze 11/A, 43124 Parma, Italy

**Keywords:** climate change, livestock welfare, alpine pastoral systems, heat stress, pasture quality, adaptation strategies

## Abstract

Extensive livestock systems in the Alps are central to biodiversity, rural livelihoods, and cultural heritage, but are increasingly threatened by climate change. This review examines how rising temperatures, altered precipitation patterns, reduced snow cover, and extreme weather events impact animal welfare in mountain farming systems. We conducted a comprehensive analysis of scientific literature addressing direct impacts such as heat stress, water scarcity, disease prevalence, and welfare-related indicators, as well as indirect effects on pasture quality, biodiversity, and socio-ecological systems. The review is based on a structured narrative search (2005–2025) across major databases (Web of Science, Scopus, and Google Scholar), using predefined keywords (e.g., climate change, welfare, Alps, ruminants, etc.) and inclusion criteria to screen ~350 studies, of which ~115 were retained and thematically synthesized. Results show that cattle are highly vulnerable to heat stress and forage decline, while sheep and goats exhibit greater resilience. However, all species are affected by reduced forage quality and increased disease risks. Climate-driven shifts in pasture composition threaten both the nutritional value and ecosystem diversity, with feedback loops that link animal welfare and biodiversity. Adaptive capacities differ across species, with goats showing the highest tolerance to resource scarcity. Potential adaptation strategies include breed selection, sustainable grazing practices, and policy frameworks that support smallholders and ecosystem services. We conclude that safeguarding animal welfare under climate change is crucial for maintaining livestock productivity, ecological sustainability, and the socio-economic resilience of Alpine communities. This review provides a foundation for developing integrated adaptation strategies that align welfare, biodiversity, and policy objectives.

## Simple Summary

Climate change is having a rapid and profound impact on the Alps, where traditional livestock farming plays a vital role in food production, biodiversity, and local culture. Rising temperatures, reduced snow cover, and more extreme weather are disrupting the grazing systems on which cattle, sheep, and goats depend. These changes reduce the quality and quantity of mountain pastures, increase heat stress, limit water availability, and promote the spread of parasites and diseases. Our review examined how these pressures affect animal welfare and compared the ability of different species and breeds to adapt. We identified that cattle are particularly vulnerable to heat stress, while sheep and goats show greater resilience, especially in harsh environments. However, all species are affected by declining forage and water resources. Possible solutions include selecting more heat-tolerant breeds, adjusting grazing practices, and developing policies that support small farmers in adopting sustainable strategies. By identifying these challenges and opportunities, our study highlights how protecting animal welfare under climate change is not only essential for livestock health and productivity but also for preserving Alpine communities, landscapes, and food traditions.

## 1. Introduction

Extensive livestock production systems are characterised by low-input, high-nature-value farming practices, which are particularly significant for maintaining biodiversity, sustaining rural livelihoods, and preserving cultural heritage [1,2,3,4]. The Alpine bioregion, spanning several European countries, supports a diverse range of livestock species, including cattle, sheep, and goats, which graze on mountain pastures during the summer months. This traditional form of animal husbandry contributes to local economies and promotes sustainable land management practices [5]. However, climate change poses increasing challenges to the viability of extensive production systems, threatening their environmental and socio-economic benefits.

Climate change has emerged as one of the most pressing global challenges of the 21st century, with wide-ranging impacts on ecosystems, human societies, and agricultural systems. At the global scale, similar climate-related vulnerabilities of livestock systems have been documented in very different socio-ecological contexts. In tropical Brazil, bioclimatic zoning based on the temperature humidity index (THI) reveals that climate change is driving increasingly severe heat stress conditions and substantial prospective milk losses in dairy herds [6]. In arid northern Mexico, bioclimatic indexes applied to confined dairy and beef cattle, pigs, and sheep reveal that projected warming will markedly increase the frequency and intensity of heat stress episodes, threatening productivity unless targeted mitigation measures are adopted [7]. At a broader regional scale, in north sub-Saharan Africa, an integrated assessment of heat stress, feed availability and water accessibility demonstrates that climate change is already constraining cattle-based milk production and that shifting herd composition towards more heat- and drought-tolerant species such as goats and camels can sustain milk yields while reducing resource use and greenhouse gas emissions [8]. These examples suggest that the impacts investigated here for the Alpine region are part of a broader global pattern of climate risk to livestock production, underscoring the importance of regional analyses in informing adaptation strategies and public policies.

The Alpine bioregion is particularly vulnerable to climate change, experiencing more rapid temperature increases than the global average [9]. Rising temperatures, changing precipitation patterns, and more frequent extreme weather events will likely disrupt livestock production systems by altering pasture productivity, increasing heat stress, and exacerbating water scarcity [10]. These environmental changes pose serious threats to animal welfare, a critical component of sustainable livestock production. Despite the importance of this issue, there is still limited research on how climate change affects livestock welfare explicitly in the extensive production systems of the Alps.

This paper aims to investigate the effects of climate change on livestock welfare in the extensive production system of the Alps. The key objectives are: (1) to assess how climate change is impacting welfare indicators such as heat stress, water availability, and welfare-related pathologies; (2) to examine the quality of the nutritional contribution of pasture modified by climate change, both in production timing and in the composition of herbaceous plants; (3) to compare the vulnerability and adaptive capacities of different livestock species (cattle, sheep, and goats) and breeds; (4) to identify adaptation strategies and policy recommendations that could enhance the resilience of Alpine livestock systems.

The primary research question guiding this study is: how does climate change affect livestock welfare in the extensive production systems of the Alps, and what adaptation strategies can be implemented to mitigate these impacts? Indeed, the complexity of the topic does not allow us to provide exhaustive answers. Still, we have attempted to outline some key aspects that we have acquired and consolidated to address this challenge best.

## 2. Review Methodology

This review was conducted following a structured narrative approach to ensure transparency and reproducibility. Scientific literature was searched using Web of Science, Scopus, and Google Scholar. Searches were conducted with a temporal focus on studies published over the last two decades (2005–2025), reflecting the period during which climate-related impacts on livestock production have been most extensively documented. The search strategy combined keywords and Boolean operators such as: “climate change” AND “livestock”, “heat stress”, “welfare” AND (“dairy cattle” OR “beef cattle” OR “sheep” OR “goat” OR “ruminants”), “Temperature–Humidity Index”, “mountain regions”, and “Alps”. Additional keyword combinations were tested iteratively to capture emerging terms in the field.

Studies were included if they addressed the following: (i) historical or projected climatic trends relevant to Alpine or mountain areas; (ii) impacts of heat stress on livestock welfare, physiology and productivity; or (iii) species-specific resilience or adaptive traits. Only peer-reviewed articles, scientific reviews, and authoritative institutional reports were considered. Non-scientific materials and studies not related to livestock or mountain environments were excluded.

The initial search yielded approximately 350 publications. After removing duplicates and screening titles and abstracts, ~170 papers were evaluated in full, and ~115 met the inclusion criteria. Reference lists of key papers were examined to identify additional relevant sources.

Extracted information was synthesized thematically, grouping evidence into climate dynamics, species-specific heat stress responses, and adaptive capacities. Climate data and bioclimatic indicators from authoritative datasets (e.g., ClimateEngine) were used to complement and contextualize the reviewed literature.

## 3. The Alpine Bioregion: Livestock Sector in the Context of Climate Change

### 3.1. Livestock Activities in the Alps

The Alps constitute one of the most emblematic and ecologically significant mountain systems in Europe, forming a vast arc that extends for about 1200 km across eight countries, from France and Monaco in the west to Slovenia in the east, encompassing Switzerland, Italy, Liechtenstein, Austria, and southern Germany. This mountain range, with peaks reaching 4810 m at Mont Blanc, covers a surface area of nearly 190,000 square kilometers and acts as Europe’s “water tower”, feeding major rivers such as the Rhine, Rhone, Po, and Danube.

Extensive livestock systems have long been integral to the Alpine region, offering economic and ecological benefits. These systems are characterised by the seasonal vertical transhumance of livestock between valley pastures and high-altitude grazing lands, a practice that supports biodiversity and prevents landscape abandonment [11,12]. Cattle, sheep, and goats are the predominant species, each adapted to different ecological niches and playing distinct roles in Alpine ecosystems. The socio-economic significance of extensive systems is particularly pronounced in marginal areas [2], where they contribute to rural livelihoods and the preservation of cultural heritage [13,14].

**Figure 1:**
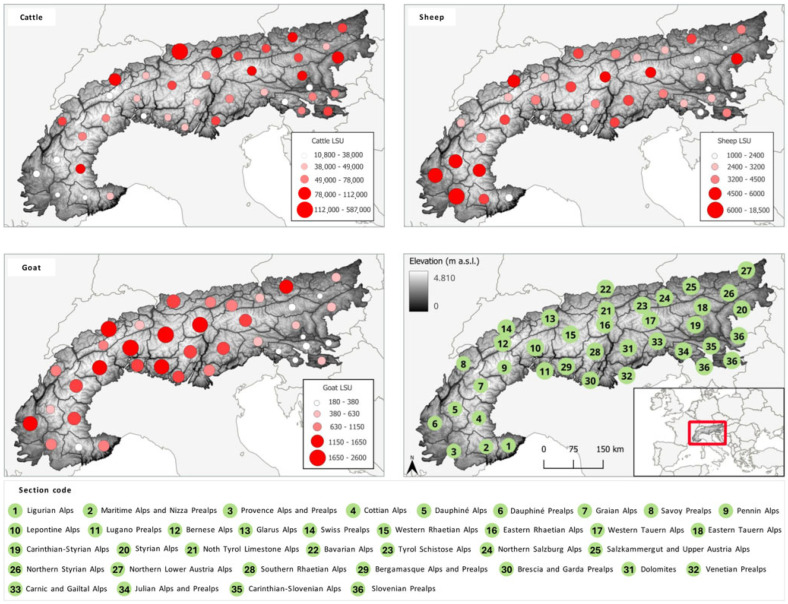
Distribution of cattle, sheep, and goat densities (LSU) in the Alpine subregions.

The distribution of cattle, sheep, and goat density in the 36 Alpine subregions is presented in Figure 1.

The livestock population in the Alpine bioregion was represented through a thematic map developed using QGIS version 3.40.5-Bratislava [15]. The analysis considered the boundaries of the Alpine arc and its subdivision into sections based on the classification proposed by Capleymar-Homoalpînus [16]. Data on livestock numbers were obtained from the database by Malek et al. [17], referring to the year 2020, which was expressed in Livestock Units (LSU). This harmonization is necessary since some countries report total cattle numbers, while others provide disaggregated data by category. Livestock data were spatially aggregated within the section boundaries through spatial intersection. Some variables in the original database contained missing values, particularly regarding goat populations in France, Germany, and Slovenia. To ensure completeness and comparability across countries, we applied the following procedure. For France, missing municipal-level goat numbers were retrieved from the national Agreste database [18]; where data were censored, values from the 2010 census were used as substitutes. In Germany and Slovenia, goat numbers are reported only at the national level, while other livestock data are available at more detailed administrative scales (districts and administrative units, respectively). In these two cases, we estimated the number of goats in the Alpine areas by allocating the national goat population proportionally to their share of the total national ruminant population located within the Alpine region. To compute these proportions, the animals numbers from Eurostat were converted into Livestock Units (LSU) following standard conversion factors [19]. The resulting goat shares were 0.19% for Germany and 0.37% for Slovenia [20].

The spatial distribution of ruminants in the Alps reveals a clear dominance of cattle over sheep and goats. Cattle livestock units (LSU) reach very high values, exceeding 100,000 LSU in several alpine sections, such as the Swiss Plateau and Tyrolean regions. Sheep are also widespread but at substantially lower densities, with maxima rarely surpassing 18,000 LSU, concentrated in the Western and Southern Alps. Goat numbers are the weakest among the three species, with most regions hosting fewer than 2000 LSU. This gradient reflects the structural role of cattle farming in alpine dairy and meat production systems. Sheep occupy marginal and higher-elevation pastures, contributing to traditional transhumance and maintaining the landscape. Goats remain localized, particularly in the Western Alps and Ligurian–Provençal areas, where they exploit steep and rocky terrain.

Using the standard Eurostat/FAO LSU factors, it is possible to convert Livestock Units (LSU) into the number of individual animals [19]. According to data from Malek et al. [17], there are approximately more than 2.6 million cattle in the Alps, followed by fewer than 1.5 million sheep. Goats are among the less common ruminant species in the Alpine region, with just over 300,000 individuals. In the Alpine region, the number of farms has decreased sharply from approximately 570,000 in 1980 to around 260,000 by 2010, with the most significant losses occurring among small holdings. This reflects a long-term structural change in mountain farming, even as livestock numbers have declined more moderately and consolidated into fewer holdings [21]. From 2010 to 2020, alpine regions in member countries exhibited similar trends. In France’s Auvergne–Rhône–Alpes region, agricultural holdings decreased from 62,694 to 48,493 (approximately 23%) [22]. Austria’s agricultural and forestry holdings fell by 11% to 154,953, with larger average herd sizes indicating consolidation [23]. Germany’s livestock holdings dropped from 144,000 to 108,000 (−25%), reflecting a shift to “fewer, but larger” farms [24]. Slovenia also experienced a decrease in farm numbers, while the average number of livestock units per holding increased from 5.6 to 6.0 [25,26]. Switzerland showed the slowest change, with 48,344 farms in 2022, marking a 1.1% decline [27]. At the European scale that underpins these alpine trends, the total LSU declined only slightly, from approximately 119 million in 2010 to approximately 113 million in 2020, implying that animal populations were more resilient than farm numbers [28].

The observed trends in livestock numbers across the Alpine region reflect long-term structural and economic transformations rather than direct climate-driven responses. Cattle remain the dominant species due to the historical centrality of dairy production, favourable market conditions and subsidy schemes, and the consolidation of increasingly specialised dairy systems. In several areas, the expansion of beef-oriented cow–calf systems, characterized by larger and more extensively managed herds, has also contributed to the recent growth in cattle numbers. At the same time, the reduction in the number of small farms and the increase in average herd size have affected both cattle and small ruminants. In sheep and goat farming, small and often mixed flocks have declined, while larger meat-oriented flocks have become more common. The contraction of dairy-oriented small ruminant farms is influenced not only by economic factors but also by the rewilding of mountain environments and rising predation pressure, particularly from the expanding wolf population. Together, these processes explain the predominance of cattle and the recent evolution of livestock populations in the Alps, independently of their differing sensitivities to climate stress.

### 3.2. Socio-Economic Relevance of the Agri-Food Chain in the Alps

Beyond its striking geomorphology, the Alpine territory constitutes a distinctive socio-economic system shaped by centuries of human settlement, transhumance, and adaptation to high altitudes environments [29]. Approximately 14 million permanent residents are dispersed across valleys, small villages, and alpine towns, where a delicate equilibrium persists between traditional primary sectors, small-scale processing industries, and a dynamic service economy driven mainly by tourism, attracting over 120 million visitors annually.

Within this context, the agri-food sector assumes a pivotal role, serving not only as a source of income but also as a cornerstone of cultural identity, biodiversity conservation, and rural livelihoods. Extensive livestock farming, based on the seasonal migration of cattle, sheep, and goats between valley farms and high mountain pastures [30], remains a central component of Alpine Agriculture. This transhumant system is crucial for maintaining open landscapes, preventing forest encroachment, and conserving high-nature-value grasslands. Characterised by low-input, biodiversity-rich practices, it stands in contrast to the intensification trends prevalent in much of European agriculture. As noted by Sturaro et al. [12] and the Alpine Convention [31], such systems are particularly significant in marginal and remote areas where alternative agricultural models are neither viable nor ecologically appropriate. The sector is sustained predominantly by small family farms and community-based cooperatives, which coordinate milk collection, cheese production, and marketing under stringent quality and origin designations [32,33].

Despite significant environmental constraints, the economic contribution of this agri-food chain remains substantial. An estimated 400,000 farms support up to 600,000 people engaged in livestock farming, dairy processing, and associated artisanal activities, generating an estimated annual value of €15–20 billion [34]. The majority of this output is derived from high-quality dairy products, notably protected designation of origin (PDO) cheeses such as Gruyère, Comté, and Fontina, as well as cured meats, alpine honey, and herbal derivatives. In many valleys, dairy production alone accounts for over half of agricultural output, underpinning both the viability of rural communities and the perpetuation of local identity.

Nevertheless, this socio-economic model is increasingly vulnerable to climate-induced pressures. The Alpine region has experienced a mean temperature rise of about 2 °C since the late 19th century, nearly double the global average [9,10,35]. This warming trend, coupled with altered precipitation regimes, reduced snow cover, and more extreme weather events, has significant implications for livestock farming, including shortened grazing seasons, declining pasture quality, the proliferation of parasites and vector-borne diseases, and heightened risks of heat stress and water scarcity. These dynamics jeopardize animal welfare and the long-term sustainability of traditional agro-pastoral systems, which rely on stable ecosystems and predictable seasonal cycles.

The scientific literature consistently highlights that the Alpine agri-food chain represents more than an economic domain; it constitutes a critical agent of ecological stewardship and cultural continuity [36,37]. It sustains biodiversity-rich habitats, maintains open landscapes, and preserves centuries-old adaptive knowledge integral to mountain life. Ensuring its resilience in the face of climate change, global market pressures, and demographic decline will require targeted policy interventions, support for smallholders, and investment in adaptive, high-value local production systems [34,38]. The long-term sustainability of the Alps thus depends not only on conserving its natural assets but equally on preserving the human and agro-ecological networks that define it as a living, rather than abandoned, landscape.

### 3.3. Climate Change in the Alps

**Figure 2:**
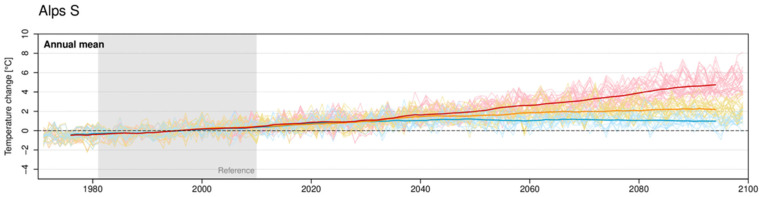
Simulated evolution of the mean annual temperature anomaly [°C] with respect to the 1981–2010 mean value (Reference period, grey area) and averaged over the Southern Alps (Alps S). Line colors refer to the different emission scenarios (for details, see [39]). Licensed under CC BY 4.0, https://creativecommons.org/licenses/by/4.0/, accessed on 8 December 2025.

The Alpine bioregion is experiencing above-average rates of climate change, with temperature having increased by approximately 2 °C since the late 19th century (+1.8 °C since 1880; [39]), nearly double the global average (Figure 2) [9,35]. This warming, which has accelerated since the 1980s, has led to intensified snow and glacier melt, shifts in precipitation patterns, and an increase in the frequency of extreme weather events [35,39,40]. These climatic changes are expected to have profound consequences for Alpine ecosystems, including alterations in vegetation patterns, water availability, and the length of the grazing season [40,41]. Despite marked local variability due to the region’s complex topography and orography [42], all Alpine ecosystems are recognized as highly sensitive to climate change [40].

Building on these observed trends, future projections indicate further warming, changes in precipitation regimes [35,40], and significant reductions in snowfall of up to 45% [43], particularly at lower elevations, where rain is increasingly replacing snow [44]. Snow cover is expected to diminish, especially below 1000 m a.s.l., while glacier volume could decline by up to 90% [40]. Moreover, extreme weather events are anticipated to become more frequent and intense [35,40], and wildfires are expected to become more widespread [45], with spatially differentiated impacts, particularly along the north–south gradient [46].

**Figure 3:**
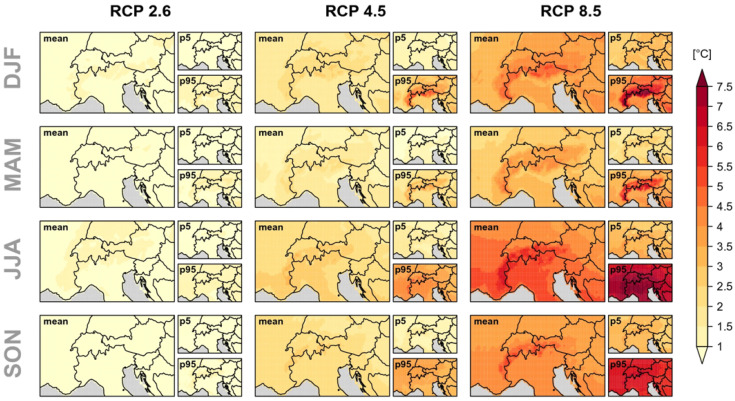
Projected seasonal mean temperature change between 1981–2010 and 2070–2099 over the Alpine region for three greenhouse gas emission scenarios (for details, see [39]). Licensed under CC BY 4.0, https://creativecommons.org/licenses/by/4.0/, accessed on 8 December 2025.

Figure 3 shows the projected spatial distribution of temperature changes across the Alpine region under three greenhouse gas emission scenarios, expressed relative to the 1981–2010 baseline. The maps indicate a consistent warming signal throughout the Alps, with particularly pronounced increases during the summer (JJA) period, which is most relevant for livestock grazing at high elevations. They also highlight clear spatial heterogeneity, reflecting the complex topography and regional climatic gradients of the Alpine arc. To provide additional context to these projections, a set of baseline summer climatology maps (Figure S1) is included in the Supplementary Materials. The maps in Figure S1 illustrate the current spatial patterns of mean temperature, relative humidity, downward shortwave radiation, and temperature-humidity index (THI) in summer (JJA) for the period from 1991 to 2020. Together, these figures provide both a present-day and a future-oriented perspective on the climatic conditions that shape livestock exposure to heat in Alpine environments.

More specifically, warming is likely to be more pronounced in summer and autumn [47], and less intense in spring, especially in the southern Alps, possibly due to Mediterranean amplification. Under high-emission scenarios, temperature increases may exceed 7 °C [39].

While total annual precipitation may remain relatively stable, seasonal redistribution is expected. Winter precipitation is projected to increase, while summer precipitation may decline by up to 40%, though with more intense rainfall events [39] and increased flood risks [48].

Glacier ablation, with a documented 49% loss in ice volume between 1900 and 2011, is driven not only by rising temperatures but also by the presence of impurities, such as dust and black carbon, which lower albedo and accelerate melting. Even with drastic reductions in CO_2_ emissions, glacier retreat would continue. This is due to their current disequilibrium and the limited altitude of Alpine peaks, which hampers the upward migration of ice masses [40]. Under optimistic scenarios, glaciers are expected to lose approximately half of their current volume [49], and the stability of permafrost will also be affected [47]. These changes will significantly impact water availability, both in terms of volume and spatial distribution, due to the emergence of new glacial lakes [50].

### 3.4. Legislation Supporting Livestock Activities in the Alps

Livestock farming remains vital to Alpine communities, sustaining rural livelihoods and preserving cultural landscapes [51]. However, climate change is exacerbating already fragile conditions, necessitating targeted policy measures. The first Alpine Convention laid the basis for sustainable development [52], followed by the 2006 Declaration on Climate Change, the 2009 Climate Action Plan, and, in 2016, the creation of the Alpine Climate Board (ACB). The ACB’s Alpine Climate Target System 2050 promotes climate-neutral farming, local products, and broader use of organic and low-input practices through sustainability indicators, regional promotion, and awareness campaigns [53]. These initiatives align with EU measures, such as the “mountain product” and the CAP 2023–2027, which allocates €34 billion for environmental goals with eco-schemes supporting agroecology and organic farming [54]. Mountain areas qualify as “natural constraint” zones for special funding [54,55]. Yet, structural issues persist, area-based subsidies disadvantage smallholders using common pastures [56], and the 2003 decoupling of payments from herd size has reduced small ruminant numbers while encouraging intensification [57]. Extensive husbandry still provides key ecosystem services: sheep and goats reduce fuel loads, limit shrub encroachment, and maintain open landscapes [58]. Regional programs, such as “Obeja Bombero” in Catalonia, value these benefits but lack EU-wide coordination [57]. Meanwhile, the return of large carnivores (wolves, bears, lynx), protected under Natura 2000, adds pressure [59]. Their recovery, driven by reforestation and land abandonment, has resulted in up to 40% pasture loss in some regions, as farmers seek safer areas [57]. This abandonment reduces biodiversity, increases wildfire risks, and harms tourism. More vulnerable species, such as sheep and goats, are often replaced by cattle, resulting in declines in traditional breeds and landscapes’ homogenization [60]. Guardian dogs help reduce predation but create tensions with tourism, complicating coexistence between farming and recreation [57].

## 4. Impacts of Climate Change on Livestock Welfare

One of the most immediate impacts of climate change on livestock welfare is heat stress, which can reduce feed intake, impair reproductive performance, and increase mortality rates [10,61]. Heat stress is particularly detrimental to cattle, which have lower heat tolerance than sheep and goats [61,62]. Additionally, prolonged periods of high temperatures can exacerbate water scarcity, thereby compromising animal welfare [63,64].

Climate change also indirectly affects livestock welfare by altering the quality and availability of pastures [65]. Rising temperatures and changing precipitation patterns can reduce forage productivity and nutritional quality, thereby increasing the risk of malnutrition [66,67]. Additionally, warmer temperatures can promote the spread of parasites and vector-borne diseases, posing additional threats to animal health [68,69]. For instance, the recent outbreak of the bluetongue virus in Sardinia, Italy, has been linked to climate-induced changes in vector dynamics [70].

### 4.1. Measuring Animal Welfare in Extensive Systems

Poor animal welfare in extensive breeding systems, where animals such as large and small ruminants are raised on open pasture with limited human intervention, can lead to a range of negative consequences. These can be categorized into direct parameters that reflect poor welfare. These parameters are measurable or observable and indicate compromised animal health, behaviour, or biological functioning:

The Body Condition Score (BCS) is one of the most used indicators, reflecting an animal’s nutritional status and overall health. A consistently low BCS is typically linked to inadequate forage, parasitic infestations, and chronic illness, which are particularly common in extensive systems [71]. In small ruminants, such as goats and sheep, poor body condition may indicate long-term nutritional deficiencies, whereas in cattle, it often correlates with lower milk production and reproductive inefficiency [72].

Lameness and physical injuries are also prevalent due to rugged terrain, long walking distances to water, and inadequate hoof care. Goats and sheep are agile but still vulnerable to injuries such as footrot and abscesses, while cattle frequently suffer from lameness linked to infectious diseases and trauma [73,74]. These conditions cause pain, restrict movement, and negatively affect feeding and social behaviours.

Disease prevalence is another critical indicator of poor welfare. In goats and sheep, internal parasites such as *Haemonchus contortus* and external parasites, including lice and ticks, are widespread, leading to anemia, poor coat quality, and reduced growth [72]. Cattle are frequently affected by tick-borne diseases, including anaplasmosis and babesiosis, which may go untreated due to the lack of veterinary care. The absence of routine vaccination and deworming programs often compounds the impact of disease.

High mortality and morbidity rates, especially among young animals (lambs, kids, and calves), are also strong indicators of compromised welfare. Causes include hypothermia, starvation, exposure, predation, and infectious diseases, often exacerbated by poor maternal care and insufficient shelter [74]. Neonatal losses in sheep and goats are frequently linked to mismothering and environmental stress, while in cattle, calf mortality is often related to poor colostrum intake and diarrheal diseases.

Behavioural abnormalities provide insights into animals’ mental states. Animals raised in extensive systems with limited handling may develop chronic stress responses, including excessive vocalization, aggression, or social withdrawal [75]. These behaviours often reflect fear, pain, or poor human–animal relationships, particularly when animals are handled infrequently or roughly.

Reproductive inefficiencies are another direct result of welfare issues. Inadequate nutrition, stress, and disease can impair fertility across species. In cattle, negative energy balance and chronic illness are associated with delayed oestrus and early embryonic loss [76]. Goats and sheep also show reduced reproductive performance when body condition is poor or parasitism is uncontrolled.

The condition of the coat and skin is an easily observable welfare parameter. In all three species, signs such as alopecia, sores, and dull coats may signal malnutrition, parasitism, or skin infections [77]. Flystrike is a severe condition in sheep caused by blowfly larvae infesting soiled fleece, while mange and lice are common welfare concerns in goats.

Failure to thermoregulate due to exposure to extreme weather is common in extensive systems where shelter is often lacking. Heat stress in cattle and small ruminants is evident through panting, drooling, and reduced feed intake, while cold stress leads to shivering and clustering behavior [78]. These conditions compromise productivity and health.

Routine painful procedures such as castration, dehorning, and tail docking are often conducted without analgesia in extensive systems. This results in significant pain and stress, evidenced by behavioral and physiological changes [79]. Despite the growing emphasis on welfare, pain management remains neglected in many regions due to a lack of awareness or resources.

Ultimately, access to water and its quality are essential to overall welfare. In extensive systems, water points may be scarce or contaminated. Dehydration affects feed intake, thermoregulation, and general health in all ruminants [80].

Goats, although more water-efficient, still require consistent hydration, especially during lactation or under high temperatures (see Table 1).

In conclusion, direct parameters of poor welfare in goats, sheep, and cattle within extensive breeding systems are multifaceted and interconnected, encompassing physiological, behavioural, and health-related indicators. These include poor body condition, lameness, high disease burden, mortality, abnormal behavior, reproductive failure, skin and coat disorders, thermal stress, procedural pain, and inadequate water access.

These indicators should be evaluated together, ideally using a welfare assessment protocol (e.g., Welfare Quality^®^ protocols, OIE animal welfare guidelines) tailored to the species and management system. Monitoring these parameters helps identify areas needing improvement to enhance animal welfare in extensive systems. Recognizing and systematically monitoring these indicators is essential for designing targeted interventions that improve animal well-being and productivity. While extensive systems are often perceived as more “natural,” the lack of regular oversight can allow severe welfare issues to persist undetected. Thus, implementing low-cost, species-specific welfare assessments and promoting basic veterinary outreach services can significantly improve welfare outcomes for ruminants in such environments.

### 4.2. Impacts on Pasture Composition and Nutritional Quality

Alpine pastures, characterized by their unique ecological conditions, are increasingly vulnerable to the effects of climate change, which significantly alters their botanical composition and, consequently, their nutritional value for grazing livestock (Argenti et al., 2020 [81]). Shifts in temperature and precipitation patterns directly influence plant phenology, species distribution, and overall ecosystem productivity, leading to complex changes in the forage base available to herbivores [82,84]. Understanding the intricate relationship between climate change and alpine pasture dynamics is crucial for developing sustainable management strategies that ensure the long-term health and productivity of these valuable ecosystems. The rising temperatures observed in alpine regions have a cascading effect, accelerating snowmelt and extending the growing season, which favors the proliferation of particular plant species over others [83]. These changes in plant community structure can have profound implications for the nutritional quality of the pasture, as different plant species vary significantly in their protein, fiber, and mineral content [98].

Furthermore, altered precipitation patterns, including increased frequency of drought events, can exacerbate these effects by limiting plant growth and reducing overall forage availability. The interplay between temperature, precipitation, and plant physiology creates a dynamic environment in which the nutritional landscape of alpine pastures continually evolves [85]. These shifts ultimately affect the health, productivity, and reproductive success of grazing animals, highlighting the need for adaptive management practices that can mitigate the negative consequences of climate change.

The scientific literature reveals a complex picture of how climate change is reshaping the composition and nutritional quality of alpine pastures [99]. Studies have documented shifts in plant community composition, with some species becoming more dominant while others decline in abundance [100]. These changes are often driven by alterations in temperature and precipitation regimes, which favor certain plant functional types over others. For example, warmer temperatures may promote the growth of shrubs, leading to changes in albedo and evapotranspiration, which in turn further influence the regional climate [101]. Alterations in snow cover duration and timing can also impact plant phenology, resulting in mismatches between plant development and herbivore foraging patterns. Moreover, the invasion of non-native species, facilitated by climate change, can further disrupt existing plant communities and alter the nutritional quality of pastures [102]. The nutritional quality of alpine pastures is also being affected by rising atmospheric carbon dioxide concentrations, which can alter plant carbon-to-nitrogen ratios, reducing the protein content of forage. Increased temperatures can also impact the nutritional content of plants, potentially reducing the quality of food sources available to wildlife [87].

The changes in pasture composition and nutritional quality have significant implications for livestock production in alpine regions. As climate change alters the availability and quality of forage, livestock producers face the challenge of maintaining animal health and productivity. Decreases in forage quality can lead to reduced animal growth rates, lower milk production, and increased susceptibility to diseases [86]. In addition to the direct effects on forage quality, climate change can also exacerbate other stressors on livestock, such as heat stress and water scarcity [90,103]. The thermal stress on animals, changes in their health, physiology, and productivity, indirectly affect the availability of feed crops and water [96]. These combined effects can significantly impact the economic viability of livestock operations in alpine areas, necessitating the development of adaptive management strategies to address these challenges. These strategies may include adjusting stocking rates, supplementing animal diets, and implementing rotational grazing systems.

Furthermore, the decrease in fodder production resulting from climate change can impact the availability of feed for animals [95]. The adoption of sustainable grazing practices, such as rotational grazing, can help to maintain plant diversity and prevent overgrazing, thereby mitigating the negative impacts of climate change. Moreover, selecting livestock breeds that are well-adapted to the changing climate conditions can also enhance the resilience of livestock production systems.

### 4.3. Consequences on Agroecosystems’ Diversity

Climate change poses a significant challenge to ecosystems worldwide, influencing biodiversity and reshaping the living conditions of both wild and domesticated animals. This intersection of environmental change, biodiversity loss, and animal welfare creates a complex triad with mutual feedback. Understanding these dynamics is essential for building sustainable agricultural systems, conserving biodiversity, and promoting ethical standards of animal husbandry. Thus, environmental protection is essential for enhancing global resilience in the face of increasing uncertainties. Within the European Community, this priority has grown due to the strategic role ecosystems play in providing critical services like food production, carbon storage in soils, and biodiversity conservation [104,105].

However, climate change alters ecosystems through rising temperatures, shifting precipitation patterns, increased frequency of extreme events, and changing seasonality. These changes directly affect species distributions, habitat suitability, phenology, and community interactions [106]. Many species, particularly those with narrow ecological niches or limited mobility, face local extinction, leading to a reduction in biodiversity across various ecosystems. In agricultural landscapes, this loss is particularly evident in grasslands and alpine meadows, where species richness of plants and arthropods declines with increasing climatic stress and land-use intensification [94]. Livestock and other domesticated animals are vulnerable to climate-related stressors. Rising temperatures and heatwaves compromise thermoregulation, feeding behavior, reproduction, and immune function [88]. Water scarcity and forage decline further exacerbate physiological stress and disease susceptibility. Welfare concerns are amplified for grazing animals exposed to open environments, such as those in highland and alpine pastures [89]. Additionally, climatic shifts may affect parasite life cycles, leading to increased exposure or novel infestations, further deteriorating animal health and welfare [91].

#### 4.3.1. Interdependence of Animal Welfare and Biodiversity

**Figure 4:**
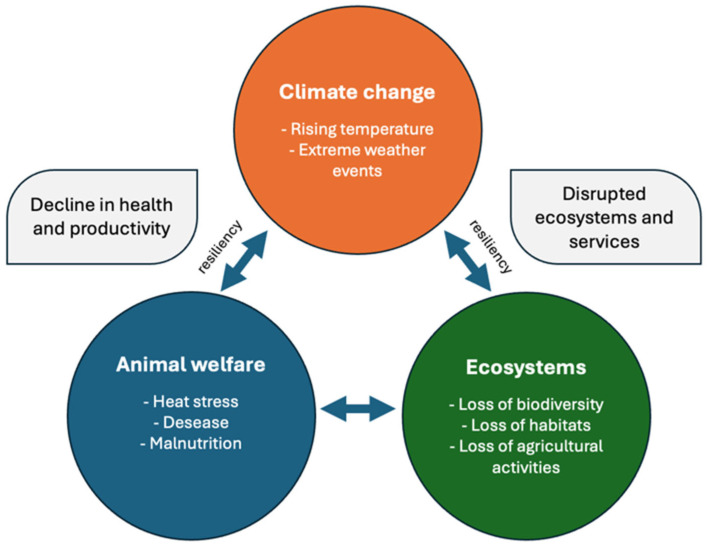
Climate-welfare-biodiversity feedback loop.

Animal welfare and biodiversity are closely linked in natural systems for different reasons (see the links depicted in Figure 4):

(i) The ecological role of livestock, such as the properly managed grazing that maintains open habitats and prevents shrub encroachment, supports diverse plant and invertebrate communities [97]. Instead of overgrazing or undergrazing that may disrupt this balance, reducing both habitat quality and animal welfare through degraded forage and exposure to harsher conditions;
(ii) Biodiversity also acts as a buffer, as high biodiversity can help stabilize ecosystems against climate variability. For example, diverse swards can provide more stable and nutritious forage across seasons, enhancing the resilience of livestock systems [107]. Rich pollinator communities also contribute to forage seed production and grassland regeneration [108];
(iii) Lately, welfare-driven biodiversity outcomes have been observed, where animals in poor welfare conditions often exhibit altered behaviors (e.g., excessive foraging, avoidance of thermal stress areas), which may change grazing pressure and spatial patterns. This can lead to heterogeneity loss and unintended ecological consequences, such as reduced insect or bird diversity [109].

#### 4.3.2. The Climate–Welfare–Biodiversity Feedback Loop

There is an emerging recognition of a feedback loop involving climate, animal welfare, and biodiversity that is organized in these terms: (a) Climate change leads to worsened animal welfare (heat stress, forage decline); (b) the compromised animal welfare affects grazing behavior, altering vegetation and soil structure; (c) these changes in turn impact biodiversity, especially insects and plants; (d) finally the loss of biodiversity can reduce ecosystem services like pollination or nutrient cycling, which are crucial for sustaining both animal health and agricultural productivity.

#### 4.3.3. Impacts of Grazing Intensity on Arthropod and Plant Biodiversity in European Pasturelands

The structure and management of European pasturelands, particularly in mountain regions such as the Alps, play a critical role in shaping plant and arthropod communities. Extensive grazing systems, often associated with low-intensity livestock management, have been shown to support higher levels of biodiversity compared to more intensive systems. These systems maintain a heterogeneous landscape that offers diverse microhabitats and floral resources, which are essential for many arthropod taxa, including pollinators [110]. Arthropod biodiversity, particularly of pollinators such as bees, butterflies, and hoverflies, is strongly influenced by the composition and structure of vegetation [111]. Extensive systems typically result in taller swards and a greater presence of forbs and flowering plants, which are associated with richer and more complex arthropod assemblages [91,112]. These environments not only provide nectar and pollen but also offer nesting substrates, shelter, and breeding habitats for invertebrates [107]. Alpine pastures and traditionally managed semi-natural grasslands are biodiversity hotspots [113], often harboring endemic species that are sensitive to both land use changes and climate warming [114].

In conclusion, the intersection between animal welfare and biodiversity conservation, particularly regarding arthropod communities in extensively grazed Alpine ecosystems, underscores the need for integrated land management strategies. These should align agricultural viability, ecological sustainability, and ethical animal husbandry to enhance the multifunctionality of pastoral systems across Europe.

### 4.4. Adaptive Capacities of Livestock Species

Different livestock species exhibit varying degrees of resilience and adaptive capacity, influenced by their physiological traits, feeding behaviors, and management practices [115]. Understanding these differences is crucial for developing sustainable livestock management strategies that can mitigate the adverse effects of climate change on Alpine agricultural systems. Cattle, sheep, and goats each possess distinct characteristics that influence their ability to cope with changing environmental conditions (see Table 2) [116].

Cattle, typically larger in size and with higher metabolic demands, may be more vulnerable to heat stress and reduced forage availability under specific climate change scenarios [117]. Their reliance on high-quality forage and susceptibility to heat stress can pose challenges in warmer and drier conditions, potentially impacting milk production and overall productivity [118]. Sheep, known for their adaptability to marginal environments, exhibit greater tolerance to water scarcity and temperature fluctuations compared to cattle [119]. Their smaller body size and efficient water utilization strategies enable them to thrive in regions with limited resources [115]. Goat transhumance, a common practice in the mountainous areas, demonstrates the adaptability of goats to challenging terrains and diverse vegetation types [120]. Goats exhibit a notable tolerance to plant secondary metabolites and a capacity to consume a diverse range of materials, enabling them to utilize regionally available byproducts and unconventional feedstuffs, which can help minimize production costs, a crucial adaptation in resource-limited environments [121].

The varied topography and microclimates within the Alpine region further contribute to the complexity of adaptation strategies. The availability of diverse forage resources at varying altitudes influences the seasonal movements and grazing patterns of livestock, necessitating adaptive management practices to optimize resource utilization and minimize environmental impacts [122].

Mountain cattle farming has undergone considerable changes over the past two decades, characterized by a decline in farm numbers, an increase in average farm size, and a weakening of the traditional link between livestock and grasslands [12,13,123]. Climate change introduces additional challenges, such as altered vegetation phenology, increased frequency of extreme weather events, and shifts in disease vector distribution. These changes can directly impact livestock health and productivity, requiring proactive adaptation measures to maintain sustainable production systems. Small ruminants can act as a buffer to environmental fluctuations, reducing financial risks for farmers [115].

From a welfare point of view, the species react differently to heat waves or water shortage. The cumulative effects of multiple stressors, such as excessive heat load, poor nutrition, and the need to walk long distances to source feed and water, can compromise production and reproduction in livestock [124].

**Table 2:** Comparative adaptive capacities of livestock ruminants in the Alpine bioregion.

Dimension		Cattle		Sheep		Goats
Heat tolerance	- - *	High susceptibility to heat stress [117,125]	+	Better tolerance to temperature fluctuations [115,119]	+ +	Well-adapted to heat and variable climates [115,121]
Water efficiency	-	Higher water needs per unit biomass and lower dehydration tolerance [61,126]	±	Intermediate water efficiency [115,127]	+ +	Adapted to arid, water-scarce conditions [127,128]
Forage requirements	- -	Bulk grazers needing higher-quality grasses; less able to utilize browse [61,129]	+	Utilizes diverse forage efficiently; some browsing flexibility [130,131]	+ +	High flexibility; tolerant of low-quality, diverse and byproduct feeds, shrubs and woody plants [132,133]
Feeding behavior	±	Primarily grazers (grass-focused); require managed pastures [129,134]	+	Intermediate grazers/foragers with moderate selectivity [130]	+ +	Browsers, tolerate plant toxins, strong ability to switch diets [130,132]
Terrain Adaptability	-	Limited to gentler terrain [135,136]	+	Can graze in marginal areas [135,137]	+ +	Thrive in steep, rocky terrain [135,136]
Climate Resilience	-	Higher vulnerability to heat/drought extremes [126,138]	+	Moderate resilience under variability; still impacted by severe droughts [115,139]	+ +	Greater resilience to combined stressors: heat, drought, low-quality feed [133,140]
Role in ecosystem	±	Grass-dominant grazing; can maintain meadows but less shrub control [136,137]	+	Maintains pasture and supports biodiversity; moderate shrub/forb use [135,137]	+ +	Controls shrubs, maintains open landscapes [121,132]
Management flexibility	- -	Needs intensive management (housing, water, feed) [61,141]	+	Suitable for extensive and marginal systems [115,139]	+ +	Highly adaptable via transhumance and low-input systems [115,128]
Economic resilience	-	High economic risk under climate stress [118,123]	+	Generally resilient economics with diversified products (meat, wool) [115,139]	+	Cost-effective in resource-limited systems; niche products [128,132]
Contribution to sustainability	±	Needs integration with ecological services; potential grassland maintenance benefits with careful stocking [126,136]	±	Supports productivity & ecological conservation; Lower input use and ability to use marginal land [115,137]	+	Use of shrubs reduces encroachment; efficient on marginal land; strong landscape services [132,137]

* Adaptative level: - -, very low; -, low; ±, moderate; +, good; + +, very good.

Cattle are more susceptible to heat stress compared to sheep and goats, potentially leading to reduced milk production and reproductive performance [125]. Implementing appropriate housing and shading strategies, along with providing access to ample water, can help mitigate the negative impacts of heat stress on cattle welfare.

Regarding feeding behaviour, the species also present significant differences. Sheep, with their selective grazing habits, can efficiently utilize a diverse range of forage resources, thereby contributing to effective pasture management and biodiversity conservation. Goats, renowned for their browsing behavior, play a vital role in controlling shrub encroachment and preserving open landscapes in mountainous regions. Ruminants can efficiently convert fibrous feedstuffs, unsuitable for human consumption, into valuable and nutrient-dense food [142]. On the contrary, cattle and sheep require nuanced management approaches that seek to optimize both economic viability and the provision of essential ecological services that healthy rangelands provide [134].

Finally, it is also important to note that the Alpine region hosts a remarkable diversity of autochthonous livestock breeds, which have evolved under harsh mountain conditions and exhibit valuable adaptive traits such as robustness to thermal stress and efficient use of marginal pastures. These local genetic resources represent an important asset for enhancing the climate resilience and long-term sustainability of Alpine livestock systems [143,144].

## 5. Conclusions

This study examines the impacts of climate change on livestock welfare in the extensive production system of the Alps, highlighting challenges such as heat stress, pasture degradation, and water scarcity, which particularly affect cattle and sheep due to their lower adaptive capacities compared to goats. These findings underscore the vulnerability of extensive production systems to climatic variability and the need for adaptive strategies.

Promising interventions include genetic selection for heat-tolerant animals, improved feeding management, and technological innovation such as precision livestock farming applications. However, significant gaps exist in understanding the socio-economic dimensions of adaptation, emphasizing the need for future research to integrate these factors. The study also addresses a geographical gap by focusing on the specific challenges in mountainous environments.

This review suggests that breeding programs should prioritize resilience in cattle and sheep and calls for participatory approaches that engage local communities in adaptation strategies, utilizing traditional knowledge. Collaborative partnerships among researchers, policymakers, and stakeholders can enhance the effectiveness of climate adaptation measures.

Future research should adopt an interdisciplinary approach, combining biophysical, socio-economic, and technological perspectives, and should include longitudinal studies to assess long-term impacts of climate change on livestock welfare.

## Figures and Tables

**Table 1:** Effects of climate change on animal performance, health, and welfare. × refer to parameter object of observation and measurement reported in the references indicated.

Climate Change Impact	Description	Effect on Animal Performance & Welfare											Animal Species Affected	Reference(s)
		Performance	Health											
			Poor Body Condition Score	Lameness & Injuries	High Disease Prevalence	High Mortality & Morbidity	Behavioral Abnormalities	Reproductive Inefficiencies	Coat and Skin Conditions	Thermal Stress (Heat/Cold)	Painful Procedures Without	Poor Water Access/Quality		
Altered plant phenology and forage quality	Decreased nutritional value of pasture affecting health, reproduction, and growth	×	×			×	×	×	×				Grazing livestock (sheep, cattle)	[81,82,83]
Drought and altered precipitation patterns	Reduced forage availability and quality; increased stress and disease vulnerability	×	×	×	×	×	×	×		×		×	Grazing livestock	[84,85,86]
Increased temperatures and CO_2_	Reduced protein content in plants; diminished food quality	×	×			×		×	×	×			Wild herbivores and livestock	[87]
Heat stress	Impaired thermoregulation, reduced feed intake, lower productivity, compromised immune function	×	×		×	×		×		×			Cattle, sheep	[88,89,90]
Increased parasite burden due to warmer climates	Greater exposure to novel or more persistent parasitic infections	×	×		×	×			×				Grazing livestock	[91]
Declining biodiversity and arthropod populations	Reduced pollination, forage regeneration, and ecological resilience	×	×										Pollinator dung beetles, livestock indirectly	[92,93,94]
Fodder scarcity and nutritional deficits	Reduced growth, lower milk production, increased disease susceptibility	×	×		×	×	×	×	×				Cattle, sheep	[95,96]
Habitat degradation from poor grazing management	Loss of plant diversity and forage quality; higher welfare risks	×	×		×	×	×	×	×				Cattle, sheep	[89,97]

## Data Availability

The original contributions presented in this study are included in the article/Supplementary Materials. Further inquiries can be directed to the corresponding author.

## Supplementary Materials

The following supporting information can be downloaded at: https://www.mdpi.com/article/10.3390/ani15243578/s1, Figure S1: Spatial distribution of air temperature (T_air_), relative humidity (RH), downward shortwave radiation (DSR), and Temperature-Humidity Index (THI) over the Alpine region for the JJA period 1991–2020 (WMO climatological normal), with the orographic profile overlaid. References [145,146,147] are cited in the Supplementary Materials.
