# Supplementary material for: Climate Change and Livestock Welfare in the Alps: A Comprehensive Review
Source: Animals (Basel). 2025 Dec 12;15(24):3578. doi: 10.3390/ani15243578 (PMC12729432; doi:10.3390/ani15243578)
Supplement: Supplementary file 1 [file animals-15-03578-s001.zip › animals-3967809-supplementary.pdf]

Figure S1 Spatial distribution of air temperature (T_{air}), relative humidity (RH), downward shortwave radiation (DSR), and Temperature-Humidity Index (THI) over the Alpine region for the JJA period 1991–2020 (WMO climatological normal), with the orographic profile overlaid.

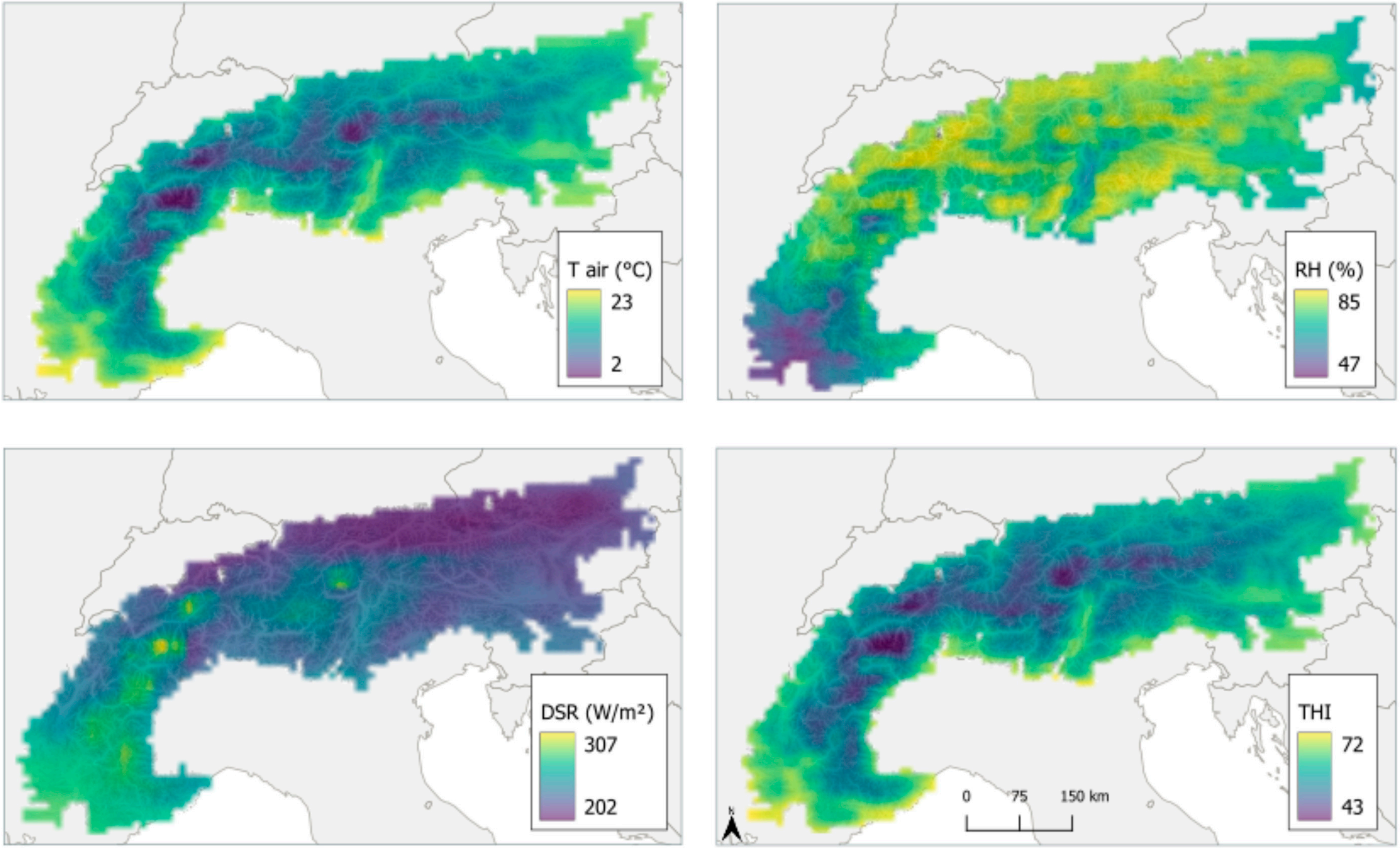

Supplementary Material

The average data for the main climatological variables were represented cartographically with reference to the climatological normal for the period 1991–2020, specifically for the boreal summer season (JJA: June–July–August). Specifically, air temperature (T_{air}), relative humidity (RH), downward shortwave radiation (DSR), and the Temperature-Humidity Index (THI) were considered. The calculations were performed using the ERA5-Ag database, which has a spatial resolution of 9600 m (Boogaard et al., 2020), downloaded via the Climate Engine platform (Climate Engine, 2025; Huntington, 2017).
RH was calculated from the dew point temperature (T_d) and T_{air} according to Equation S1, using Tetens' equations (Equation S2) to calculate the vapor pressure above water ($T_{air} \geq 0$ °C) and above ice ($T_{air} < 0$ °C) (Murray, 1967).
The THI was calculated using Equation S3 proposed by Silva et al. (2020). Data on T_d and wind speed (W_s) used were also obtained from the ERA5-Ag database using the same methods described above.

$$RH = 100 \cdot \frac{e_s(T_d)}{e_s(T_{air})} \quad \text{(S1)}$$

$$e_s\left(T_{(d,\ air)}\right) = \begin{cases} 0.61078 \cdot e^{\left[\frac{17.27 \cdot T_{(d,\ air)}}{T_{(d,\ air)} + 237.3}\right]}, if\ T_{(d,\ air)} \geq 0\,°\,C \\ 0.61078 \cdot e^{\left[\frac{21.875 \cdot T_{(d,\ air)}}{T_{(d,\ air)} + 265.5}\right]}, if\ T_{(d,\ air)} < 0\,°\,C \end{cases} \quad \text{(S2)}$$

$$THI = (6.3952 + 0.08964 \cdot T_{air} + 0.01018\ W_s)^2 \quad \text{(S3)}$$

where RH is relative humidity (%); $e_s(T_d)$ is the vapor pressure at the dew point temperature (kPa); $e_s(T_{air})$ is the vapor pressure at the air temperature (kPa); T_d and T_{air} are the dew point temperature and the annual mean air temperature, respectively; THI is the Temperature Humidity Index; W_s is the annual mean wind speed ($m \cdot s^{-1}$).

References

1. Climate Engine, version 2.1. Desert Research Institute and University of California, Merced. 2025. Available online: http://climateengine.org (accessed on 20 November 2025).
2. Huntington, J.; Hegewisch, K.; Daudert, B.; Morton, C.; Abatzoglou, J.; McEvoy, D.; Erickson, T. Climate Engine: Cloud Computing of Climate and Remote Sensing Data for Advanced Natural Resource Monitoring and Process Understanding. Bulletin of the American Meteorological Society. 2017. Available online: https://journals.ametsoc.org/view/journals/bams/98/11/bams-d-15-00324.1.xml (accessed on 20 November 2025).
3. Boogaard, H.; Schubert, J.; De Wit, A.; Lazebnik, J.; Hutjes, R.; Van der Grijn, G. Agrometeorological indicators from 1979 to present derived from reanalysis. Copernicus Climate Change Service (C3S) Climate Data Store (CDS). 2020. Available online: https://cds.climate.copernicus.eu/datasets/sis-agrometeorological-indicators?tab=overview (accessed on 20 November 2025).
